# Targeting Tumor Adaption to Chronic Hypoxia: Implications for Drug Resistance, and How It Can Be Overcome

**DOI:** 10.3390/ijms18091854

**Published:** 2017-08-25

**Authors:** Jae-Young Kim, Joo-Yong Lee

**Affiliations:** Graduate School of Analytical Science and Technology (GRAST), Chungnam National University, Daejeon 34134, Korea; jaeyoungkim@cnu.ac.kr

**Keywords:** chronic hypoxia, cancer, tumor, HIF-1α, drug resistance

## Abstract

The rapid and uncontrolled proliferation of tumors limits the availability of oxygen and nutrients supplied from the tumor vasculature, thus exposing them to low oxygen environments. Thus, diminished oxygen availability, or hypoxia, is the most common microenvironment feature of nearly all solid tumors. All living cells have the ability to sense changes in oxygen tension and adapt to this stress to preserve survival. Likewise, cancer cells adapt to chronic hypoxic stress via several mechanisms, including promotion of angiogenic factor production, metabolic shift to consume less oxygen, and reduction of apoptotic potential. Adaptation of tumor cells to hypoxia is believed to be the main driver for selection of more invasive and therapy-resistant cancer phenotypes. In this review, we discuss molecular mechanisms by which tumor cells adapt to hypoxia, with a specific focus on hypoxia-inducible factor (HIF) transcription factor. We further discuss the current understandings on hypoxia-mediated drug resistance and strategies to overcome it.

## 1. Introduction

Tumor cells require a constant supply of oxygen. In small tumors with a diameter less than 1 mm, oxygen can be supplied by diffusion from blood vessels. However, as the tumor grows, cells distant from the blood vessel are exposed to limited oxygen availability, leading to the formation of necrotic tumor tissue. A fraction of cells which survive this hypoxic stress become problematic, since they exhibit a more invasive phenotype and are refractory to cancer therapies [1,2,3]. Thus, targeting hypoxia is becoming an exciting area garnering significant attention in the efforts to overcome cancer drug resistance. In this review, we discuss our recent understandings on molecular mechanisms by which hypoxic tumor cells adapt to hypoxic stress, with a focus on hypoxia-inducible factor (HIF) transcription factor and current therapeutic strategies to attenuate hypoxia-mediated drug resistance.

### 1.1. Hypoxia-Inducible Factor (HIF)

#### 1.1.1. HIF Regulation in Hypoxia

**Figure 1:**
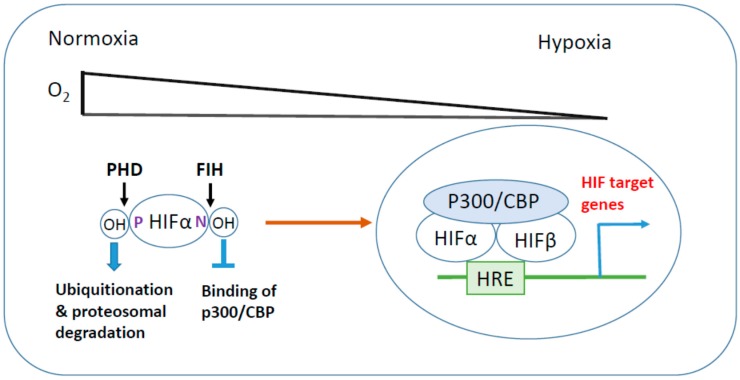
Hypoxia-inducible factor (HIF) activation in hypoxic stress. Under normoxic conditions, HIF-α is hydroxylated at its critical proline (P) and arginine (N) residues by prolyl hydroxylase domain (PHD) and factor inhibiting HIF (FIH), respectively, leading to proteosomal degradation and suppression of transcriptional activity. In response to hypoxic stress, inhibitory hydroxylations of HIF-α are reduced, then HIF-α is stabilized and translocates to the nucleus where it heterodimerizes with HIF-β. HIF-α/β dimer associates with transcriptional coactivator p300/CBP and binds to hypoxia response element (HRE) to induce HIF target gene expression.

The HIF family of transcription factor plays a pivotal role in the adaptation of cancer cells to hypoxia. The HIF transcription factor functions as a heterodimer, composed of one of two oxygen-labile α subunits (HIF-1α, -2α) and an oxygen-insensitive β subunit. HIF-1α is expressed ubiquitously, whereas HIF-α is expressed only in particular cell types such as hepatocytes and endothelial cells [4]. The stability of HIF-α family proteins are tightly regulated by oxygen status. Under normoxic conditions, a set of enzymes called HIF prolyl hydroxylase domain family proteins (PHDs) hydroxylates two critical proline residues in the HIF-α subunits. On hydroxylation of the critical proline residues, the von-Hippel Lindau (VHL) tumor suppressor E3 ligase recognizes and ubiquitinates HIF-α, leading to rapid proteosomal degradation. In addition to the proline residues, HIF-α subunits contain a critical arginine residue in the C-terminal transactivation domain. Factor inhibiting HIF (FIH) hydroxylates HIF-α at these arginine residues, leading to decreased transcriptional activity via disruption of its binding with transcriptional coactivator p300/CREB-binding protein (CBP). Since both PHD and FIH require oxygen as a substrate, the hydroxylation of HIF-α is impaired under hypoxic conditions. This leads to the stabilization and translocation of HIF-α to the nucleus where it heterodimerizes with HIF-1β. The HIF-α/β heterodimer complex binds to the hypoxia-responsive elements (HRE) located in the promoter region of HIF target genes. Through this mechanism, HIF transcription factors promote the expression of target genes involved in angiogenesis, metabolic adaptation, migration, and suppression of apoptosis [5,6,7] (Figure 1).

#### 1.1.2. HIF Expression in Tumors

The robust growth of tumor cells creates a relatively large distance between a portion of the tumor area and the tumor vasculature. Due to limited diffusion of oxygen from the blood vessels (which has been measured to be around 150 µm [8,9]), some tumor cells are inevitably exposed to hypoxic stress. The resulting limited supply of oxygen to tumor cells leads to the formation of centric regions of necrotic cells, which are frequently observed upon histological examination of human solid tumors [10]. This makes hypoxia one of the most common features within the microenvironment of solid tumors, and hypoxia responsive HIF-1α level is found to be particularly elevated in many human solid tumors including colon, gastric, lung, and prostate cancers [11]. Although hematological malignancies are not considered solid tumors, recent studies revealed the unique hypoxic environment of bone marrow promoting maintenance of hematological cancer stem cells. These studies indicate that hypoxia–HIF axis could play a pivotal role in the development of hematological malignancies and drug resistance as well [12,13,14]. HIF-1α overexpression is also mediated by aberrant activation of oncogenic signaling providing a mechanism for adaptation to hypoxia by tumor cells, in addition to hypoxia-mediated mechanisms. V-Src, but not c-Src, promotes HIF-1 expression and its target gene vascular endothelial growth factor (VEGF) expression [15]. HIF-1 expression is induced by activation of the oncogenic receptor tyrosine kinases, including epidermal growth factor receptor (EGFR), fibroblast growth factor receptor (FGFR), and insulin-like growth factors 1/2 receptor (IGF1/2R) [16]. Of note, HIF-1 is required for the expression of IGF2 and IGF-binding proteins (IGFBP)-2 and -3, suggesting that HIF-1 promotes the autocrine growth factor loop [16].

### 1.2. Mechanism of Tumor Adaptation to Hypoxia, and Its Implication in Drug Resistance

#### 1.2.1. Hypoxia-Induced Autophagy

Hypoxia-induced autophagy is now recognized as a part of adaptive mechanisms promoting cell survival. Autophagy is an evolutionally conserved cellular process that clears old or damaged cellular components. Although all cells undergo basal autophagy under normal conditions, autophagy is induced by stresses such as nutrient starvation, metabolic stresses, and hypoxia in order to maintain metabolic homeostasis [17]. Since such stresses are common in nutrient deficient solid tumors, autophagy is recognized as a key regulator of cellular viability in cancer cells. The precise role of autophagy in cancer development is still elusive; however, increasing evidence indicates that autophagy is associated with poor outcome in multiple cancers as well as with therapy resistance, indicating the cytoprotective role of autophagy protecting cancer cells [18,19,20,21,22]. Ablation of autophagy augments the efficacy of chemotherapeutic reagents [18,23,24] as well as targeted reagents in imatinib-treated chronic myeloid leukemia [25] and vemurafenib-treated *BRAF* mutant melanoma [26]. The cytoprotective role of autophagy is presumably mediated by the recycling of ATP and cellular breakdown products to maintain cellular biosynthesis and survival [27]. Given its importance in drug resistance, autophagy could be involved in the failure of cancer therapies associated with hypoxia, and targeting autophagy could therefore be an attractive option to improve therapy outcomes. Like other hypoxic responses, HIF transcription factor plays a key role in hypoxia-induced autophagy induction. Bellot and colleagues reported that the HIF-1α target genes, *BNIP3* and *BNIP3L*, are responsible for the induction of autophagy under hypoxic conditions via disruption of the Bcl-2:Beclin1 complex, and HIF-mediated autophagy is a survival mechanism involved in tumor progression [28]. Attenuation of HIF-1 expression inhibited hypoxia-induced autophagy and potentiated the efficacy of cytotoxic treatment under hypoxia, supporting the probable role of HIF in hypoxia-induced drug resistance [29]. Of note, it has been reported that HIF-1 specifically promotes hypoxia-induced autophagy of mitochondria, namely mitophagy, leading to the downregulation of oxidative phosphorylation during metabolic adaption of cancer cells to hypoxia. Hypoxia-induced mitophagy prevents the accumulation of reactive oxygen species (ROS), thereby promoting survival, via induction of HIF-dependent transcription of the *BNIP3* gene [30]. However, this notion needs to be supported by future studies, wherein the following points need to be clarified: (i) the physiological role of hypoxia-induced mitophagy in the context of drug resistance; and (ii) the importance of HIF-induced BNIP3 and/or BNIP3L in hypoxia-induced drug resistance. While physiological hypoxia-induced autophagy is recognized as a survival mechanism, autophagy associated with severe hypoxic condition (O_2_ level less than 0.1%), namely anoxia, could lead to a different outcome. Anoxic condition is often accompanied by the drastic restriction of nutrients (e.g., amino acids, glucose), leading to inactivation of the mammalian target of rapamycin (mTOR) pathway as a result of AMP-activated protein kinase (AMPK) activation [31]. Impaired mTOR activity is linked to induction of autophagy, and this HIF-independent autophagy induction under severe hypoxia is reportedly associated with autophagic cell death, rather than cytoprotection [32]. Thus, targeting hypoxia should be carefully considered when treating tumors which are likely to be exposed to severe hypoxic condition.

#### 1.2.2. Regulation of Cell Death under Hypoxia

Under hypoxic conditions, the non-adapted cancer cells undergo apoptosis, which provides for the strong selection of cells that survive cancer therapy. Hypoxia induces apoptosis, which is dependent on HIF-1 and p53-dependent mechanisms [33]. HIF-1 reportedly induces transcription of the Bcl2-family proteins, BNIP3 and NIX, promoting apoptosis under hypoxic condition [34,35,36]. BNIP3 is a pro-apoptotic mitochondrial protein through interaction with E1B19K and Bcl2 [37]. Interestingly, BNIP3-mediated cell death is independent of cytochrome C release and caspase activation. Rather, it involves increased plasma and mitochondrial membrane permeability, leading to mitochondrial damage and mitophagy. This is followed by loss of mitochondrial electrochemical potential and increased production of reactive oxygen species (ROS), which is a typical phenotype of aponecrosis [38,39]. However, other studies report that HIF-induced BNIP3 is poorly apoptotic, thereby the proapoptotic role of BNIP3 in hypoxia is still controversial [28,40]. Hypoxia also induces mitochondrial pathway apoptosis via increasing the p53 activity. Under severe hypoxic conditions, where the oxygen levels fall below 0.2%, p53 protein is phosphorylated and accumulated via the ataxia telangiectasia and Rad3-related protein (ATR) kinase signaling pathway [41]. Also, several reports indicate that HIF-1α stabilizes p53 to induce apoptosis [42,43,44,45].

In contrast, other studies indicated that hypoxia promotes anti-apoptotic pathways under certain conditions, such as DNA damage stress. Hypoxia attenuates the expression of pro-apoptotic proteins including Bax and Bid in a HIF-1 independent manner, which contributes to chemoresistance in colon cancer cells [46]. In the context of non-small cell lung cancer (NSCLC), the expression of the anti-apoptotic protein survivin is positively correlated with HIF-1α, and promoter activity for survivin expression is impaired by mutating the HIF-1α binding site, thus indicating that hypoxia promotes survival signaling via HIF-1α-survivin axis [47]. Moreover, the hypoxia protects mammary epithelial cells from anoikis-induced cell death by blocking the expression of pro-apoptotic proteins Bim and Bmf [48]. Survivin is reportedly a mediator of doxorubicin resistance in breast cancer [49], suggesting hypoxia-induced survivin expression could promote chemoresistance in at least some human solid tumors. Here, one importance question is raised; what is the molecular mechanism differentiating apoptosis-resistant and -sensitive cells under hypoxic stress? Dong and colleagues reported that hypoxic induction of the inhibitor of apoptosis protein-2 (IAP-2) promotes the survival of cells under hypoxic stress [50,51], raising the possibility that cells overexpressing IAP family proteins are resistant to hypoxia-induced apoptosis. As IAP family proteins have been implicated in the development of cancer development [52,53], it is worth further investigating the precise role of IAPs in the hypoxic adaptation of cancer cells, and, furthermore, in the development of drug resistance. Overall, the effect of hypoxia and subsequent activation of HIF-1 transcription factor in the determination of cell fate is multifaceted and context dependent (e.g., related to tumor type and oxygen concentration). It is plausible that hypoxia-induced cell death drives the selection of a drug-resistant population within the tumor.

### 1.3. Strategies to Improve Therapy

#### 1.3.1. Targeting HIF-1 Directly

HIF-1 is a central mediator for the adaptation of cancer cells to hypoxia, via the aforementioned mechanisms. The genetic deletion of HIF-1 in endothelial cells (ECs) disrupts hypoxia-induced EC behavior, leading to the profound inhibition of tumor formation [54]. Several studies have reported that suppression of HIF-1 via RNAi impairs tumor progression. The in vivo repression of HIF-1 using RNAi resulted in tumor regression associated with increased necrosis [55]. Suppression of HIF-1 by RNAi, antisense oligonucleotide, and the dominant negative form of HIF-1 showed anti-tumor effects in various preclinical models including pancreatic cancer, tongue squamous cell carcinoma, and gastric cancer [56,57,58]. Thus, targeting the HIF-1 activation has emerged as an attractive therapeutic strategy to inhibit tumor progression, and to potentially overcome drug resistance. However, although the genetic modulation of HIF-1 expression (e.g., via RNAi) is not yet generally applicable in the clinic, small molecule inhibitors targeting HIF-1 could be an attractive way to overcome the adversity of HIF-1. HIF-1 chemical inhibitors could be divided into two groups: (i) agents modulating HIF-1 transcriptional activity; and (ii) agents modulating the HIF-1 expression. For transcriptional activation, HIF-1 forms a protein complex with transcriptional coactivator p300. Through high-throughput screening, Kung and colleagues identified chetomin as a disruptor of the HIF:p300 binding. Chetomin disrupts the structure of CH1 domain of p300, which is required for its association with HIF-1, thereby leading to the attenuation of hypoxia-inducible transcription and the inhibition of tumor growth in the xenograft prostate cancer model [59]. Echinomycin, a cycle peptide with anti-microbial properties, reportedly binds to the HIF-1 recognition sequence 5′-CGTG-3′, and inhibits the DNA-binding and transcriptional activation of HIF-1 [60]. However, a previous phase II clinical study showed that echinomycin has minimal anti-tumor activity associated with severe side effects, limiting its application to the clinic [61]. It has been reported that several compounds are able to reduce HIF-1 levels. Geldanamycin and its analog 17-AAG are inhibitors of molecular chaperon heat shock protein 90 (Hsp90), which is required for HIF-1 protein stability [62]. Geldanamycin induces the degradation of HIF-1, accompanied by the reduction of HIF-1 transcription activity in kidney and prostate cancer cells [63]. Several clinical trials have evaluated 17-AAG in combination with kinase inhibitor drugs, including Raf inhibitor sorefenib and human epidermal growth factor receptor 2 (HER2) inhibitor trastuzumab, in VHL mutant kidney cancer and HER2-positive breast cancers, respectively [64,65]. Bortezomib, a proteasome inhibitor, is reported to inhibit tumor adaptation to hypoxia via blocking of hypoxic activation of HIF-1 and induction of its target genes, including VEGF and erythropoietin (EPO) [66]. Since inhibition of chaperone or proteasome could be highly non-specific and disrupt normal tissue functions, efforts have been made to discover more specific HIF-1 inhibitors. It is reported that PX-478 suppresses the constitutive and hypoxia-induced levels of HIF-1 in cancer cells, and exhibits an antitumor effect in human tumor xenograft model [67]. NSC-134754 was discovered from a compound screening to inhibit both HIF-1 protein levels and its activity induced by hypoxia, and subsequent induction of HIF-1 target gene expression [68,69].

#### 1.3.2. Targeting HIF-1 Signaling

In addition to targeting HIF-1, another method to reverse the adverse effects of HIF-1 would be: (i) to block signaling pathways leading to HIF-1 accumulation and activation; and (ii) to target the consequence(s) of HIF-1 activation. Studies reported hypoxia-independent induction of HIF driven by activation of kinase signaling, which could provide potential therapeutic strategies to inhibit HIF activation. The aberrant activation of PI3K-AKT-mTOR signaling induces the overexpression of HIF-1 in cancer [70], and the dual PI3K/mTOR inhibitor, NVP-BEZ235, suppresses hypoxia-induced HIF-1 expression and enhances apoptosis of cancer cells under hypoxic stress [71]. Resveratrol, a natural compound known to be anti-tumorigenic, reportedly decreases HIF-1 and its target gene (*VEGF)* by inhibiting PI3K-AKT and mitogen-activated protein kinase (MAPK) activation [72]. In addition, the Ras inhibitor *trans*-farnesylthiosalicylic acid (FTS) exhibits a profound antitumor effect in glioblastoma cells by repressing the Ras-signaling induced HIF-1 expression [73]. Fara-A, a nucleotide analog, inhibits HIF-1 expression, and blocks the VEGF transcription via the inhibition of PI3K-AKT signaling in ovarian cancer cells [74]. Moreover, the activation of receptor tyrosine kinase (RTK) is linked to HIF induction, enhancing tumor adaptation to hypoxia. Multiple RTK signaling, which includes VEGFR, platelet-derived growth factor receptor (PDGFR), and EGFR, increase the HIF-1 and HIF-2 expression, and therefore the pharmacological inhibition of these RTKs using tyrosine kinase inhibitors (TKIs) could abrogate hypoxia-induced HIF accumulation in the context of neuroblastoma cells [75]. Interestingly, some studies report that hypoxia can promote oncogenic RTK signaling, indicating a bidirectional crosstalk between oncogenic (RTK) signaling and HIF. Hypoxia-induced HIF promotes mRNA expression of an oncogenic RTK c-Met, amplifies HGF signaling, thereby promoting invasive growth of cancer under hypoxic condition [76]. This suggests c-Met is an important mediator of tumor progression under hypoxia, and this notion was supported by a study reporting that pharmacological inhibition of c-Met abrogates hypoxia-induced invasion in a mouse lung cancer xenograft model [77]. Furthermore, c-MET inhibitor PHA665753 sensitizes gastric cancer cells to radiotherapy [78]. As hypoxia is believed to be one of main drivers of radiotherapy resistance [79], it is reasonable to mention that targeting c-Met could be an attractive way to inhibit tumor progression as well as to overcome radiotherapy resistance. Hypoxia is also linked to overexpression of the ERBB family of RTKs, whose aberrant expression is closely associated with multiple human cancers [80], and with drug resistance to tyrosine kinase inhibitors (TKIs) targeting ERBB proteins. EGFR/ERBB1 expression can be induced in response to hypoxia via the HIF-2α dependent increase of EGFR mRNA translation, providing a non-mutational activation of EGFR expression in human tumors [81]. Thus, in HER2/ERBB2-driven breast cancer, hypoxia-induced HIF-1 promotes HER2 inhibitor lapatinib resistance via inhibiting MAPK phosphatase dual-specificity phosphatase (DUSP2) expression, thereby compensating the loss of MAPK activity in lapatinib-treated cells [82]. These studies indicate that HIF-1 can be induced by aberrant activation of oncogenic RTKs, and hypoxia-induced HIF activation promotes RTK expression, indicating the possible existence of a positive feedback loop. Hence, a combinatory approach that attacks this loop could be effective, especially in cancers driven by oncogenic RTK activation. Finally, *VEGF* is a well-established HIF-1 target gene associated with tumor progression via the promotion of hypoxia-induced angiogenesis. The efficacy of anti-VEGF monoclonal antibody bevacizumab (Avastin) has been evaluated in multiple clinical studies, showing good clinical effects when combined with chemotherapy in breast, non-small cell lung cancer, renal cell carcinoma, pancreatic cancer, and sarcoma, whereas the effect of monotherapy is marginal [83,84,85].

#### 1.3.3. Targeting Hypoxia-Induced Autophagy

Given the importance of autophagy in tumor adaption to hypoxia and its implication in hypoxia-associated drug resistance, targeting tumor specific autophagy would be an attractive way to improve cancer therapy. The key step in autophagy is the fusion of autophagosomes with acidic lysosome, where the autophagosome is degraded and recycled back to the cytosol [86]. The increased glycolysis in a hypoxic tumor results in tumor acidity, which is closely linked to tumor progression and drug resistance [87,88]. Hence, acidic organelles such as lysosomes play a key role in processes under hypoxia, and agents that inhibit endosomal acidification, including an antimalarial drug chloroquine and proton pump inhibitors (PPIs), are proposed as possible anticancer strategies. A known PPI, pantoprazole, inhibits autophagy possibly through inhibiting the acidification of endosomes and then autophagosome fusion [89]. Tan and colleagues reported that autophagy is a mechanism of docetaxel resistance, and inhibiting autophagy by pantoprazole enhanced the efficacy of docetaxel [90,91]. Several other studies indicate that PPIs such as omeprazole and esomeprazole also overcome chemoresistance via inhibition of autophagy [92,93,94]. 

#### 1.3.4. Targeting Hypoxia-HIF to Improve Immunotherapy

**Figure 2:**
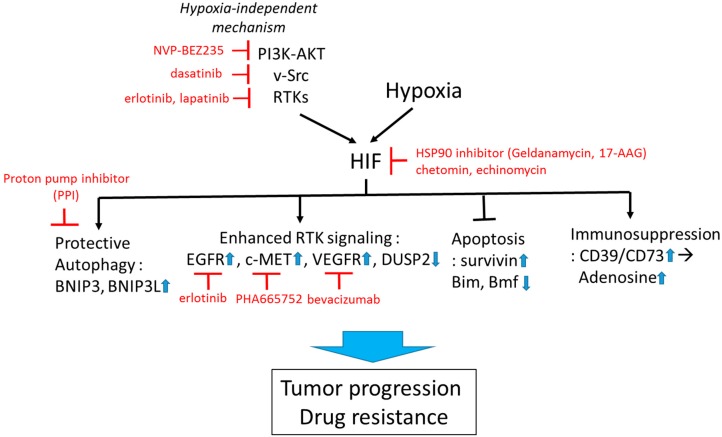
Targeting HIF to overcome hypoxia-associated drug resistance. HIF can be induced by both hypoxia-dependent and -independent mechanisms. Active HIF in tumor cells promotes drug resistance via upregulation of cytoprotective autophagy, receptor tyrosine kinase (RTK) signaling, suppression of apoptosis, and promotion of immunosuppressive tumor microenvironment. Examples of pharmacological approaches targeting HIF-regulating and -regulated mechanisms are shown in red. Small blue arrows indicate the direction of changes of protein abundance or activity after HIF activation.

Recent evidence indicates that chronic tumor hypoxia is linked to tumor maintenance via the suppression of T lymphocytes, thus targeting hypoxia-HIF axis has emerged as a novel therapeutic approach to improve immunotherapy. HIF-dependent expression of CD39/CD73 ectoenzymes is responsible for the accumulation of extracellular adenosine in tumor microenvironment. The resulting activation of adenosine receptors (A2AR/A2BR) elevates cAMP level in T cells leading to inhibition of anti-tumor T cell function, providing tumor permissive tumor microenvironment [95,96]. Hatfield and colleagues showed that exposing tumor bearing mice to hyperoxia (60% oxygen) decreased intratumoral hypoxia and concentration of extracellular adenosine via decreasing CD39/CD73 expression in tumor, reversing hypoxia-adenosinergic immunosuppression [97,98]. These studies indicate that the suppression of HIF signaling could target cancer cells as well as relieve hypoxia-mediated immunosuppressive mechanisms (Figure 2).

## 2. Summary

Despite recent advances in anti-cancer therapies, cancer still remains the leading cause of death worldwide. Drug resistance is the primary cause for cancer therapy failure, and its underlying mechanisms involve pharmacokinetic resistance, tumor cell intrinsic resistance, and factors associated with tumor microenvironment. The relatively overlooked importance of microenvironment-induced drug resistance has recently been recognized. Hypoxia is a common feature of the microenvironment of many solid tumors and hematological malignancies, and increasing evidence indicates that it promotes tumorigenesis, and, furthermore, confers drug resistance by altering the tumor cell physiology in regard to the reduction of apoptotic potential, induction of cytoprotective autophagy, and immunosuppressive tumor microenvironment. Since the HIF-1 transcription factor is a central player in the hypoxic adaption of tumor cells, therapeutic strategies targeting HIF-1 itself or signaling pathways up and downstream of HIF are garnering significant attention in the effort to overcome hypoxia-induced drug resistance. Targeting tumor specific autophagy and receptor tyrosine kinase activation associated with hypoxia/HIF could be another attractive therapeutic option, however, challenges still remain. First, drugs targeting HIF lack specificity (e.g., HSP90 inhibitor) or mechanism of action, limiting clinical application of those drugs. Chemical library screenings could lead to the discovery of more specific HIF inhibitors in the near future. Second, targeting the kinase signaling network associated with HIF could lead to acquired drug resistance, allowing reactivation of HIF. The occurrence of this bypass signaling is a common feature in kinase inhibitor therapy, thus rational drug combination to abrogate cancer cells’ adaptive responses should also be considered. Third, it is important to note that the degree and significance of hypoxic adaptation to cancer therapy varies for each patient. Thus, in the era of personalized medicine, tumors need to be screened for hypoxia markers such as HIF-1 and biochemical markers for autophagy in order to achieve more specific targeted therapies to overcome hypoxia-mediated drug resistance. Overall, given the important role of hypoxia-induced drug resistance, therapeutic strategies hijacking hypoxia adaptation should be considered in combination with conventional cancer therapies.
